# Conceptualization of Heterogeneity of Chronic Diseases and Atherosclerosis as a Pathway to Precision Medicine: Endophenotype, Endotype, and Residual Cardiovascular Risk

**DOI:** 10.1155/2020/5950813

**Published:** 2020-02-12

**Authors:** Vadim V. Genkel, Igor I. Shaposhnik

**Affiliations:** Department of Internal Medicine, Federal State Budgetary Educational Institution of Higher Education “South-Ural State Medical University” of the Ministry of Healthcare of the Russian Federation, Vorovskogo St. 64, 454092 Chelyabinsk, Russia

## Abstract

The article discusses modern approaches to the conceptualization of pathogenetic heterogeneity in various branches of medical science. The concepts of endophenotype, endotype, and residual cardiovascular risk and the scope of their application in internal medicine and cardiology are considered. Based on the latest results of studies of the genetic architecture of atherosclerosis, five endotypes of atherosclerosis have been proposed. Each of the presented endotypes represents one or another pathophysiological mechanism of atherogenesis, having an established genetic substrate, a characteristic panel of biomarkers, and a number of clinical features. Clinical implications and perspectives for the study of endotypes of atherosclerosis are briefly reviewed.

## 1. The Concept of “Endophenotype”: Definition and Scope

In the classical definition of the term “endophenotype” (EP), given by Gottesman and Gould, EP is designated as “measurable components unseen by the unaided eye along the pathway between disease and distal genotype” [1]. Somewhat later in 2006, Gottesman and Gould identified six attributive signs of EP, and also defined the scope and purpose of this concept in neuroscience and psychiatry [2]. Currently, EP is defined as a genetically determined, measurable indicator with interindividual variability that is consistent with the variability of the target trait of a higher order (for example, normative function) [3, 4].

EP, occupying an intermediate position on the “genotype-EP-phenotype” pathway, can be represented at any level of analysis—anatomical, biochemical, neuropsychological, etc. Regardless, EP should reflect the genetic vulnerability of the individual and have an additional diagnostic value in comparison with the overt phenotype, since it represents a simpler and more accurate “marker” of the genetic basis of the disease [5]. However, the concept of EP is not synonymous with the concept of a biomarker. EP, unlike the biomarker, is genetically determined and is an integral part of the pathophysiological process (causally associated with it and occupying a place along the “genotype-phenotype” pathway underlying the disease [6, 7]). In other words, EP is always a biomarker, while the biomarker is not always EP [6].

In psychiatry and neuroscience, the concept of EP is used quite fruitfully. To date, various EPs of schizophrenia, autism spectrum disorders, bipolar affective disorder, etc. [8, 9] have been proposed and are being actively studied. For example, the following EP of schizophrenia can be distinguished at various levels: structural (reduction of the gray matter of the frontal and temporal lobes, reduction of the hippocampus); neurophysiological (eye-movement deficits); behavioral (introversion), etc. [10, 11].

## 2. EP Concept in Internal Medicine

The concept of EP in internal medicine and related fields has now found limited application.

In a study by Shoda et al., three EPs of eosinophilic esophagitis were identified [12]. At this, each EP had a number of distinctive features at the genetic, immunological, morphological, endoscopic, and clinical levels. It is extremely important that the definition of EP in this case may contribute to the modification of the patient's therapy, since belonging to a particular EP determined the effectiveness of steroid therapy. Manjarrez-Orduño et al. identified the EP of systemic lupus erythematosus, which, according to the authors, is observed in 30-45% of patients [13]. This EP, designated as “cytotoxic,” is characterized by an increase in the content of CD8^+^ of T-lymphocytes (CCR7-CD45R^int-hi^ CD28^−^), producing various cytotoxic mediators (interferon-*γ*, perforin, and granzymes), and a high incidence of lupus nephritis.

In 2018, the report on asthma “After asthma: redefining airways diseases” was published in The Lancet [14]. In this paper, an attempt and a call were made for the deconstruction of “asthma” as a single nosological form for various endotypes, each of which have pathophysiological mechanisms with significant differences. The historically established umbrella term “asthma” leveled the heterogeneity of this clinical syndrome, artificially combining various processes into one group. Ignoring this fundamental heterogeneity determined, on the one hand, nonoptimal planning and interpretation of clinical studies of drugs, and on the other, hindered the development of a precision medicine approach to managing patients with asthma.

The authors examined the question of how the phenotypic (clinical) heterogeneity of asthma can be explained by different pathophysiological mechanisms or endotypes [14, 15]. It was noted that today it is not fully understood how far the phenotypic heterogeneity can affect our understanding of endotypes, since many phenotypic signs (for example, symptoms) can be caused by different mechanisms. As a result, the authors chose a reductionist approach, according to which, when identifying the EP of asthma, it is proposed to focus on traits that have different developmental mechanisms which can be recognized and diagnosed, and can also serve as a therapeutic target. Thus, the following EP of asthma has been proposed: airflow limitation, airway inflammation, airway infection, impaired airway defences, and altered cough reflex sensitivity and efficacy [14, 16].

It should be noted that several endotypes of asthma have also been proposed earlier. Thus, depending on the nature of the inflammation pattern, neutrophilic and eosinophilic endotypes of asthma are distinguished [17]. Lötvall et al., in 2011, proposed the following endotypes of asthma: aspirin-associated asthma, allergic bronchopulmonary mycosis, allergic asthma, late-onset bronchial asthma (hypereosinophilic), and asthma of cross-country skiers [18]. Each of the endotypes proposed by Lötvall et al. have their own genetic basis, developmental mechanisms, characteristic biomarker profile, etc. [19].

It is worth noting that Pavord et al. and Lötvall et al. understand the concept of “endotype” somewhat differently, and accordingly, the endotypes of asthma they distinguish significantly differ. Endotypes of Pavord et al. are more consistent with the “classical” notions of EP, while the endotypes of Lötvall et al. occupy a more distal position along the “genotype-EP-phenotype” pathway, approaching directly the phenotype, that is, the disease. Moreover, these endotypes are essentially different forms of asthma (disease), and therefore, cannot fully meet the EP criteria given by Gould and Gottesman (EP must segregate with illness in the general population) [2]. Russell and Baillie define the endotype as “a subgroup within a population of patients who are distinguished by a shared disease process,” that is, with different leading mechanisms for the development of the disease [20]. This definition more closely matches the types that have been identified by Lötvall et al. In general, it is possible to define an endotype as a subtype of a disease defined by a distinctive leading pathophysiological mechanism [21].

Thus, at present, the conceptualization of the pathophysiological heterogeneity of the disease is carried out in terms of the “endophenotype” and “endotype.” Despite the differences in these approaches, each of them pursues the same utilitarian goals:

- A better understanding of endotypes of diseases will help optimize the planning and interpretation of clinical trials of new drugs and treatment methods. For example, in a clinical study of brodalumab blocking interleukin- (IL-) 17, which did not demonstrate a significant improvement in asthma, the proportion of patients with eosinophilic, rather than neutrophilic inflammation, for which brodalumab should be as effective as possible, was likely to be high [14]. Extrapolation of the results obtained from all endotypes of the disease does not contribute to the introduction of precision medicine and leads to suboptimal therapeutic regimens
- Identification of endotypes of various diseases will give impetus for the initiation of a number of studies of new or already used drugs that have targeted the leading mechanisms in this particular endotype (for example, the study of various anti-inflammatory agents (methotrexate, colchicine) in patients with atherosclerotic cardiovascular diseases)
- Identification of endotypes of diseases will optimize and personalize existing treatment regimens

## 3. The Concept of Endotypes (EPs) in Cardiovascular Medicine and Atherosclerosis Research

In cardiovascular medicine, the concept of endotypes is used mainly in studies of heart failure (HF) and hypertension. Endotypes of HF are studied quite intensively in recent years [22]. Shah et al., through the method of machine learning, identified three endotypes of HF with preserved ejection fraction, significantly differing in clinical characteristics, features of the structure and function of the heart, hemodynamics, and the clinical trajectory of patients [23]. Tromp et al., using cluster analysis, identified six endotypes of HF, differing by clinical profile, biomarker profile, prognosis, and response to therapy (response to titration angiotensin-converting enzyme inhibitors and beta-blockers) [24]. Moreover, the actual classification of HF depending on the ejection fraction, fixed by clinical recommendations, also represents different biological phenotypes or endotypes of HF [25].

Hypertension is also understood for a long time as a heterogeneous group of subtypes with different etiologies and pathogeneses [26]. A number of researchers have identified various endotypes of hypertension: low-renin hypertension, salt-and-stress-sensitive hypertension, hypertension associated with obesity, etc. [27–29].

Atherosclerosis is a complex systemic disease, the development of which is determined by interactions of genetic, epigenetic, and environmental risk factors [30]. Adequate “endotyping” of atherosclerosis is impossible without an analysis of current studies of the genetic architecture of atherosclerosis and related diseases. For the first time, Farrer proposed to isolate endophenotypes of atherosclerosis as a possible approach to the study of the genetics of atherosclerosis, based on the dissection of various endotypes that have a strong genetic component [31]. Wyszynski and Farrer identified six endotypes of atherosclerosis: an increase in low-density lipoproteins (LDL), reduced high-density lipoproteins (HDL), an increase in lipoprotein(a) (Lp(a)), an increase in blood pressure, increased homocysteine, and metabolic syndrome [32, 33]. However, over the past years, an understanding of the genetics and pathophysiology of atherosclerosis has progressed significantly, which necessitates a revision of these endotypes.

According to large meta-analyses, 57 loci have been identified that are statistically significantly associated with the development of atherosclerosis and about 100 loci have been identified that are presumably associated with atherosclerosis [34]. Xi et al., after analyzing 45,304 publications, found references to 1,312 genes associated with atherosclerosis [35]. At least half of the known loci associated with the risk of developing coronary artery disease (CAD) demonstrate pleiotropy and are associated with the risk of other diseases or pathological processes [36].

The pathways associated with atherosclerosis and their genetic substrate are conserved during phylogenesis. Therefore, according to von Scheidt et al., among the 178 mechanisms associated with ischemic heart disease in humans, 53.2% are identical to those in mice [34]. The leading mechanisms common to humans and mice include disorders of lipid metabolism, hemostasis, and immune regulation. Xi et al., using gene ontology analysis, identified 50 pathophysiological pathways involved in the development of atherosclerosis [35]. The most highly overrepresented pathway went to the inflammation and Toll-like receptor signaling pathway [35].

According to the genome-wide association study (GWAS), among the currently identified loci associated with an increased risk of CAD and myocardial infarction, 24% are associated with lipid metabolism, 10% are associated with blood pressure, 2% are associated with carbohydrate metabolism, and 4% are associated simultaneously with several components [37]. 60% of the loci were not associated with the classic risk factors for atherosclerosis, and the interpretation of their role in atherogenesis is difficult.

It should be noted that when describing individual endotypes, much attention was paid to the results of studies with Mendelian randomization and GWAS. Of course, it is necessary to take into account the limitation of these types of studies, including the difficulty of identifying the nature of detectable associations between certain genetic variants and the trait of interest [38].

Nevertheless, the results of GWAS make it possible to identify regions of the genome and gene clusters that are important in the pathogenesis of polygenic diseases, which contributes to a better understanding of the mechanisms underlying certain diseases [39].

One of the promising areas that can combine the achievements of genetics and a whole range of fundamental and clinical disciplines is the multiomics approach, which should contribute to the identification of causal links between genetic variants and the molecular mechanisms of chronic multifactorial diseases [40].

Below, we consider the possible endotypes of atherosclerosis, selected on the basis of the leading pathophysiological mechanisms that have an established genetic substrate (the data are summarized in Table 1).

### 3.1. Endotype, Associated with an Increase in LDL

#### 3.1.1. Background

The key event in the development of atherosclerosis is subintimal accumulation of ApoB-containing lipoproteins, more than 90% of which are the LDL fraction. Today, there is no doubt that an increase in the concentration of LDL-cholesterol (namely, the cumulative burden of LDL-cholesterol) is the main causal factor in the development and progression of atherosclerosis [41]. The European Atherosclerosis Society Consensus Panel “Sensitivity of low-density lipoproteins cause atherosclerotic cardiovascular disease” discusses in detail evidence of the causal role of LDL in atherosclerosis [41].

#### 3.1.2. Genetics

Currently, various variants of genetic mutations that cause an increase in LDL and the development of atherosclerosis and related cardiovascular diseases are known: LDLR, PCSK9, APOE, APOB-100, SORT1, ANGPTL3, CELSR2, PSRC1, HMGCR, etc. [42–44]. In studies using the multiomics approach, the causal effects of the genetic variants SORT1 and HMGCR have been established [45, 46].

#### 3.1.3. Biomarkers

Biomarkers reflecting the cumulative burden of LDL-cholesterol include the following: total cholesterol, LDL-cholesterol, apolipoprotein B, apolipoprotein B-100, oxidized LDL (ox-LDLs), modified LDL, small dense LDL, and concentration of PCSK9.

#### 3.1.4. Clinical Features and Comorbid Conditions

This endotype is a “reference.” Hypercholesterolemia, caused by mutations in the LDLR gene, is a classic model of atherosclerosis in experimental studies.

In the last decade, the role of dyslipidemia in the development of cognitive impairment, including Alzheimer's disease, is actively discussed. There is evidence of a common genetic substrate of dyslipidemia and Alzheimer's disease (APOE, ABCA7, and SORT1) [47, 48]. Also, according to Mendelian randomization studies, genetically determined high cholesterol is associated with an increased risk of developing breast cancer [49]. Moreover, hyperlipidemia may be associated with an increased risk of prostate and colon cancer [50, 51].

### 3.2. Endotype, Associated with an Increase in Lp(a)

#### 3.2.1. Background

Elevated levels of Lp(a) predominantly cause damage to medium and large caliber arteries, as well as the aortic valve leaflets. There are three levels of evidence for the causal role of Lp(a) in the development of atherosclerosis: epidemiological studies, Mendelian randomization studies, and GWAS. The LPA gene contains elements that determine the inflammatory response with the participation of interleukin-6, which, together with other data, may indicate a close relationship between Lp(a) and inflammation in atherogenesis [52].

#### 3.2.2. Genetics

The level of circulating Lp(a) is mainly genetically determined by the LPA gene locus and is not significantly influenced by other genetic, dietary, or environmental factors [53]. Proteomix studies have identified networks of associated proteins that confirm the atherogenic effects of Lp(a) [54]. These networks include proteins involved in the inflammatory response (complement components), platelet aggregation (platelet activating factor), and fibrinolysis.

#### 3.2.3. Biomarkers

Biomarkers associated with an increase in Lp(a) include the following: Lp(a), apolipoprotein isoform (a), and antibodies to Lp(a) [55].

#### 3.2.4. Clinical Features and Comorbid Conditions

Patients with an increase in the level of Lp(a) associated with mutations in the LPA gene have an increased risk of the development and progression of aortic stenosis [56, 57]. Also, an increase in the content of Lp(a) may be associated with an increased risk of venous thromboembolism [58]. On the other hand, an increase in Lp(a) may be associated with a decrease in the relative risk of developing diabetes mellitus [59, 60].

### 3.3. Endotype, Associated with Arterial Injury (Arterial Hypertension)

#### 3.3.1. Background

Hypertension is a proven “driver” of atherosclerosis with an established genetic substrate, which currently makes up more than 120 loci [61]. Hypertension, endothelial dysfunction, and vascular stiffness are closely interrelated and constitute an independent pathophysiological atherogenesis cluster.

Polymorphisms associated with elevated blood pressure also lead to the development of disorders in the normal physiology of the vessel: the migration of smooth muscle cells in response to damage (ADAMTS7), endothelial cell migration and neointimal repair, and remodeling of small resistance arteries [61].

#### 3.3.2. Genetics

Currently, numerous genetic loci that determine the normal physiology of the vessel have been discovered: ADAMTS7, THBS2, CFDP1, NOX4, EDNRA, PHACTR1, GUCY1A3, CNNM2, CYP17A1, etc. [61, 62].

The significant role of proteins that play a role in maintaining vascular homeostasis in atherogenesis has been demonstrated in a number of studies using proteomics technologies, for example, FBLN1C (fibulins) [63].

Mutations in these genes cause, on the one hand, the development of hypertension, endothelial dysfunction, and an increase in vascular stiffness, and on the other hand, the development of atherosclerosis.

#### 3.3.3. Biomarkers

Biomarkers associated with arterial injury include the following: endothelin, angiotensin, adrenomedullin, natriuretic peptides, von Willebrand factor, cell adhesion molecules, endothelial progenitor cells, endothelial microparticles, nitric oxide, and asymmetric dimethylarginine.

#### 3.3.4. Clinical Features and Comorbid Conditions

The states that are, in particular causally, associated with hypertension include the following: hemorrhagic stroke, chronic kidney disease, atrial fibrillation, vascular dementia, and left ventricular hypertrophy [64, 65].

### 3.4. Endotype, Associated Mainly with the Activity of Inflammation

#### 3.4.1. Background

Inflammation, the driver of which is represented by various proatherogenic lipoproteins, is considered as the central mechanism of atherogenesis [66]. CANTOS (Canakinumab Anti-Inflammatory Thrombosis Outcomes Study) confirmed the “inflammatory hypothesis” of atherothrombosis [67].

Atherogenesis in systemic inflammatory diseases (rheumatoid arthritis, systemic lupus erythematosus, etc.) proceeds at an accelerated rate regardless of the presence of classical risk factors and is determined mainly by the activity of systemic inflammation, as well as duration and phenotype of the disease [68, 69].

#### 3.4.2. Genetics

The following genes involved in the regulation of various pathways of inflammation, according to various GWAS, are significantly associated with atherosclerotic cardiovascular diseases (CVD): CXCL12, MCP-1, TLRs, SH2B3, HLA, IL-6R, IL-5, PECAM1, and others [70]. In studies based on the proteomics approach, the crucial role of inflammation was also shown by the example of the activation of the TNF-*α* pathway in the vascular wall, as well as the migration of monocytes into the subendothelial compartment (ANRIL) [71, 72].

Metabolomics studies have demonstrated strong relationships between the level of metabolites reflecting the activity of the inflammatory response, such as N-acetylneuraminic acid and lactate, and the progression of atherosclerosis [73].

Moreover, mutations in key genes determining cellular interactions of immunocompetent cells cause, on the one hand, the development of atherosclerotic CVD, and on the other, systemic inflammatory diseases, such as rheumatoid arthritis [74]. These key genes include the HLA gene (HLA-DRB1∗04), TNF, and IL-1 [74].

#### 3.4.3. Biomarkers

Biomarkers associated mainly with the activity of inflammation include the following: TNF, IL-1b, IL-6, IL-12, IL-18, IL-23, IFN-g, IL-17, IL-22, TH17 cells, hsCRP, pentraxin-3, sCD40L, VCAM, and ICAM [75].

#### 3.4.4. Clinical Features and Comorbid Conditions

This endotype is most pronounced in chronic systemic inflammatory diseases such as rheumatoid arthritis, systemic lupus erythematosus, psoriasis, and ankylosing spondylitis. The severity and speed of atherosclerosis development in rheumatoid arthritis and systemic lupus erythematosus are similar to those in diabetes mellitus [76, 77]. The relative risk of carotid plaque detection in patients with systemic lupus erythematosus is 2.4 times higher than in the general population, and the maximum risk (5.6 times) is observed in patients younger than 40 years old [78]. In patients with chronic inflammatory diseases, phenotypically unstable plaques are found more frequent with less stenosing of the lumen of the vessels [79].

Among the comorbid conditions associated with chronic inflammatory diseases, the high frequency of solid tumors (in the prostate, mammary glands, uterus, and skin), mental disorders (depression and psychosis), and osteoporosis should be noted [80, 81].

### 3.5. Endotype, Associated with Metabolic Risk Factors

#### 3.5.1. Background

Diabetes mellitus and a cluster of metabolic risk factors (hyperglycemia and obesity) have been considered for many years as an independent predictor of atherosclerosis. Diabetes mellitus implements proatherogenic effects through a variety of mechanisms: hyperglycemia, insulin resistance, reduced nitric oxide bioavailability, oxidative stress, systemic inflammation, etc. [82].

#### 3.5.2. Genetics

According to GWAS, 24% of the loci associated with the development of diabetes also increase the risk of atherosclerosis [83]. Genetic variants associated with diabetes independently increase the risk of CAD, taking into account gene pleiotropy and general pathophysiological pathways (dyslipidemia, hypertension, etc.) [84]. In addition, studies using the metabolomics approach have demonstrated strong relationships between the levels of D-glucose, 1,5-anhydrosorbitol, D-mannose, and myoinositol, and the development of atherosclerosis [73].

Currently, seven variants are known that are common elements in the genetic architecture of diabetes and atherosclerosis: TCF7L2, HNF1A, CTRB1/2, MRAS, ZC3HC1, MIR17HG, and CCDC92 [84].

#### 3.5.3. Biomarkers

Biomarkers associated mainly with metabolic risk factors include the following: blood glucose, blood insulin, C-peptide, glycated hemoglobin, glycated albumin, sRAGE, fructosamine, etc. [85].

#### 3.5.4. Clinical Features and Comorbid Conditions

Diabetes contributes to the development of medial calcinosis. Medial calcinosis, in turn, significantly limits “positive” vessel remodeling in response to plaque growth, which probably leads to an increase in the frequency of occlusive diffuse polyvascular diseases in diabetes [82, 86]. Moreover, in small caliber arteries (for example, the tibial arteries), the phase of “positive” remodeling may be absent, which leads to early development of occlusive lesion. Diabetes is probably a protective factor in the development of abdominal aortic aneurysm [87].

Among the comorbid conditions associated with a cluster of metabolic risk factors, the following are included: psoriasis, Alzheimer's disease, affective disorders, and cancer (colorectal, pancreas, breast, and kidney) [88, 89].

## 4. Сoncluding Remarks

We have a few comments on the proposed endotypes of atherosclerosis. First, we did not include in our proposed model an endotype associated with an increase in the level of homocysteine. The causal role of homocysteine in the development of atherosclerosis and CVD has not yet been proven and, in general, data on the role of homocysteine are contradictory. According to GWAS, which included 44,147 people, genetic variants that affect plasma homocysteine concentrations are not associated with the risk of CAD [90]. The results of large-scale studies with Mendelian randomization also do not confirm the causal role of homocysteine in the development of atherosclerotic CVDs [91].

Secondly, the value of HDL in the development of atherosclerosis has also not been established to date. The protective role of an increase in HDL and the proatherogenic effects of a decrease in HDL are subject to reasonable doubt [92]. Research data with Mendelian randomization also do not confirm the causal role of HDL in the development of atherosclerotic CVDs [93].

Thirdly, the hemostatic system plays an extremely important role in vascular biology and atherogenesis. However, according to currently available data, the role of the hemostasis system is realized mainly in the late stages of atherogenesis, determining the prognosis of patients who are already clinically manifesting atherosclerotic CVD [94]. In this regard, we also did not propose an endotype, associated mainly with disorders in the hemostasis system.

## 5. Clinical Implications and Perspectives

In recent years, the concept of residual cardiovascular risks has been actively developed, designed to facilitate the study of the mechanisms of adverse cardiovascular events in patients with atherosclerotic CVD receiving optimal medical therapy [95]. To date, a number of researchers have identified lipid, inflammatory, thrombotic, and metabolic residual risks [96]. Residual risk associated with an increase in Lp(a) is also considered [97].

According to Patel et al., the current guideline-based approach is not patient-specific enough and does not allow influencing the causal mechanisms leading to the progression of atherosclerotic CVD [96]. The concept of residual risks in this case should contribute to the recognition in patients of different pathophysiological mechanisms, of which the contribution to the progression of CVD is a leading one, and the impact on which will bring maximum benefit to the patient.

Thus, conceptualization of the pathogenetic heterogeneity of atherosclerosis and atherosclerotic CVDs at the late stages of atherogenesis occurs. Paradoxically, understanding this heterogeneity at the stages of initiation and development of atherosclerosis develops rather slowly. However, specific interventions in the early stages of atherosclerosis can be much more effective and beneficial for both the patient and the health care system as a whole.

Studies using the omics approach are extremely promising in this context. Moreover, these studies today provide examples of early individualized diagnosis of chronic cardiometabolic diseases, which is a significant progress in precision medicine [98].

Further study of the genetics of cardiovascular diseases and the use of approaches based on system biology and machine learning will probably make it possible to make substantial progress in this area in the near future.

## Figures and Tables

**Table 1:** Summary of the main endotypes of atherosclerosis [36, 37, 42, 61, 62, 70, 84, 93, 99–105].

Endotype	Genetics	Biomarkers	Clinical features and comorbid conditions
LDL	LDLR, PCSK9, APOE, APOB-100, SORT1, and ANGPTL3	Total cholesterol, LDL-C, ApoB, ApoВ-100, ox-LDLs, modified LDL, sdLDL, and PCSK9	Alzheimer's disease, breast cancer, colon cancer, and prostate cancer
Lp (a)	LPA	LP(а) and apo(a) isoforms	Aortic stenosis and venous thromboembolism
Hypertension	ADAMTS7, THBS2, CFDP1, NOX4, EDNRA, PHACTR1, GUCY1A3, CNNM2, CYP17A1, FGF5, and NOS3	Endothelin, angiotensin, adrenomedullin, natriuretic peptides, von Willebrand factor, cell adhesion molecules, endothelial progenitor cells, endothelial microparticles, NO, and asymmetric dimethylarginine	Hemorrhagic stroke, chronic kidney disease, atrial fibrillation, vascular dementia, and left ventricular hypertrophy
Inflammation	MCP-1, M-CSF, VCAM-1, IL-6R, PECAM1, SH2B3, CXCL12, SMAD3, and TLRs-genes	TNF, IL-1b, IL-6, IL-12, IL-18, IL-23, IFN-g+IL-17+IL-22+TH17 cells, hsCRP, pentraxin-3, sCD40L, VCAM, and ICAM	Rheumatoid arthritis, inflammatory bowel disease, solid tumors, and psychiatric disorders
Metabolic	HNF1A, SH2B3, PPP1R3B, and CTRB1/2	Blood glucose, blood insulin, C-peptide, glycated hemoglobin, and glycated albumin	Diabetes mellitus, maturity onset diabetes of the young, hepatic adenoma, psoriasis, Alzheimer's disease, affective disorders, breast cancer, colorectal cancer, and pancreatic cancer

Comments: EDNRA=endothelin receptor type A; PHACTR1=phosphatase and actin regulator 1; GUCY1A3=guanylate cyclase 1 soluble subunit alpha 1; CNNM2=cyclin and CBS domain divalent metal cation transport mediator 2; CYP17A1=cytochrome P450 family 17 subfamily A member 1; SORT1=sortilin 1; FGF5=fibroblast growth factor 5; HNF1A=hepatocyte nuclear factor 1 homeobox A; SH2B3=SH2B adaptor protein 3; PECAM1=platelet endothelial cell adhesion molecule 1; CXCL12=C-X-C motif ligand 12; ANGPTL3=angiopoietin-like protein 3; PPP1R3B=protein phosphatase 1 regulatory subunit 3B; CTRB1/2=chymotrypsinogen B1; SMAD3=mothers against decapentaplegic homolog 3; NOS3=nitric oxide synthase 3; LDLR=low-density lipoprotein receptor; PCSK9=proprotein convertase subtilisin/kexin type 9; APOB=apolipoprotein B; sdLDL=small dense low-density lipoproteins; ADAMTS=a disintegrin and metalloproteinase with thrombospondin motifs; THBS2=thrombospondin-2; CFDP1=craniofacial development protein 1; NOX4=NADPH oxidase 4; MCP-1=monocyte chemoattractant protein 1; M-CSF=macrophage colony-stimulating factor; VCAM-1=vascular cell adhesion molecule 1; IL-6R=interleukin-6 receptor; HNF1A=hepatocyte nuclear factor 1 homeobox A; TNF=tumor necrosis factor; IL=interleukin; hsCRP=high-sensitivity C-reactive protein; sCD40L=soluble CD40 ligand.
